# Bis{6-meth­oxy-2-[(4-methyl­phen­yl)iminiometh­yl]phenolate-κ^2^ *O*,*O*′}tris­(nitrato-κ^2^ *O*,*O*′)holmium(III) mono­hydrate

**DOI:** 10.1107/S1600536810047227

**Published:** 2010-11-20

**Authors:** Jin-Bei Shen, Guo-Liang Zhao

**Affiliations:** a College of Chemistry and Life Sciences, Zhejiang Normal University, Jinhua 321004, Zhejiang, People’s Republic of China; b Zhejiang Normal University Xingzhi College, Jinhua, Zhejiang 321004, People’s Republic of China

## Abstract

The crystal structure of the title compound, [Ho(NO_3_)_3_(C_15_H_15_NO_2_)_2_]·H_2_O, contains two Schiff base 6-meth­oxy-2-[(4-methyl­phen­yl)iminiometh­yl]phenolate (*L*) ligands, three independent nitrate ions that chelate to the Ho^III^ ion with O atoms and a hydrate water mol­ecule. The coordination environment of the Ho^III^ ion is ten-coordinate. The *L* ligands chelate with a strong Ho—O(phenolate) bond and weaker Ho—O(meth­oxy) contacts. The latter can be inter­preted as the apices of the bicapped square–anti­prismatic [HoO_10_] polyhedron. Inter­molecular N—H⋯O hydrogen bonds occur. Intra­molecular O—H⋯O inter­actions link the complex mol­ecules and uncoordinated water mol­ecules.

## Related literature

For the crystal structure of a zinc(II) complex with the same the same ligands as in the title compound,, see: Xian *et al.* (2008 ▶). For the crystal structure of a terbium(III) complex related to the title compound, see: Zhao *et al.* (2007 ▶). For an ytterbium(III) complex, see: Liu *et al.* (2009 ▶). For a zigzag chain cadmium(II) complex bridged by chloride, see: Li *et al.* (2008 ▶). For iron(III) and cobalt(III) complexes of some *N*-salicyl­idene­amino acids in the form of a powder, see: Burrows & Bailar (1966 ▶). For the syntheses of rare earth complexes with Schiff base ligands derived from *o*-vanillin and adamantane­amine, see: Zhao *et al.* (2005 ▶).
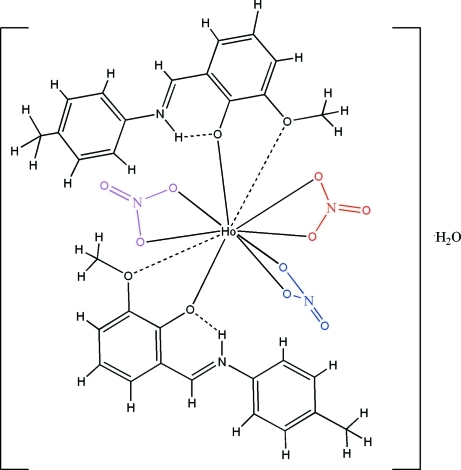


## Experimental

### 

#### Crystal data

[Ho(NO_3_)_3_(C_15_H_15_NO_2_)_2_]·H_2_O
*M* *_r_* = 851.54
Triclinic, 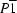

*a* = 9.7646 (4) Å
*b* = 9.9813 (4) Å
*c* = 18.4281 (11) Å
α = 97.862 (3)°
β = 101.688 (3)°
γ = 106.270 (2)°
*V* = 1652.21 (14) Å^3^
*Z* = 2
Mo *K*α radiation
μ = 2.47 mm^−1^
*T* = 296 K
0.3 × 0.2 × 0.1 mm

#### Data collection

Bruker APEXII area-detector diffractometer
Absorption correction: multi-scan (*SADABS*; Sheldrick, 1996 ▶) *T*_min_ = 0.558, *T*_max_ = 0.781
20799 measured reflections
5775 independent reflections
5426 reflections with *I* > 2σ(*I*)
*R*_int_ = 0.025

#### Refinement

*R*[*F* ^2^ > 2σ(*F* ^2^)] = 0.021
*wR*(*F* ^2^) = 0.059
*S* = 1.05
5775 reflections
455 parameters
H-atom parameters constrained
Δρ_max_ = 0.53 e Å^−3^
Δρ_min_ = −0.67 e Å^−3^

### 

Data collection: *APEX2* (Bruker, 2006 ▶); cell refinement: *SAINT* (Bruker, 2006 ▶); data reduction: *SAINT*; program(s) used to solve structure: *SHELXS97* (Sheldrick, 2008 ▶); program(s) used to refine structure: *SHELXL97* (Sheldrick, 2008 ▶); molecular graphics: *SHELXTL* (Sheldrick, 2008 ▶); software used to prepare material for publication: *SHELXL97*.

## Supplementary Material

Crystal structure: contains datablocks I, global. DOI: 10.1107/S1600536810047227/om2370sup1.cif

Structure factors: contains datablocks I. DOI: 10.1107/S1600536810047227/om2370Isup2.hkl

Additional supplementary materials:  crystallographic information; 3D view; checkCIF report

## Figures and Tables

**Table 1:** Selected bond lengths (Å)

Ho1—O2	2.2734 (18)
Ho1—O3	2.2787 (18)
Ho1—O12	2.388 (2)
Ho1—O10	2.414 (2)
Ho1—O7	2.418 (2)
Ho1—O6	2.435 (2)
Ho1—O13	2.483 (2)
Ho1—O9	2.519 (2)
Ho1—O4	2.742 (2)
Ho1—O1	2.803 (2)

**Table 2:** Hydrogen-bond geometry (Å, °)

*D*—H⋯*A*	*D*—H	H⋯*A*	*D*⋯*A*	*D*—H⋯*A*
O1*W*—H1*WA*⋯O10^i^	0.88	2.11	2.996 (6)	177
O1*W*—H1*WB*⋯O11^ii^	0.88	1.94	2.817 (6)	177
N1—H111⋯O2	0.86	1.98	2.655 (3)	135
N2—H222⋯O3	0.86	1.88	2.588 (3)	138

Symmetry codes: (i) 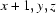; (ii) 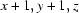.

**Fig. 1.**
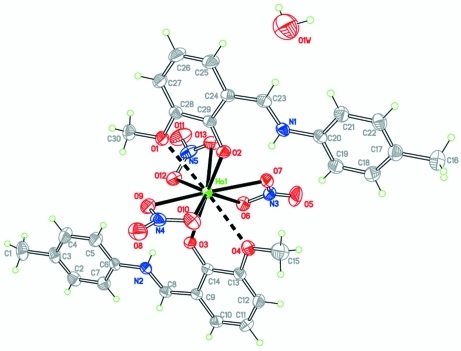
The molecular structure of the title complex, showing the atom-labeling scheme. Displacement ellipsoids are drawn at the 30% probability level.

**Fig. 2.**
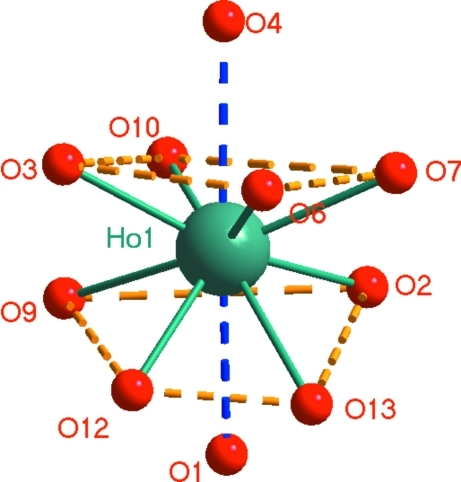
The coordination environment of the holmium(III) atom, showing the bicapped square antiprism.

**Fig. 3.**
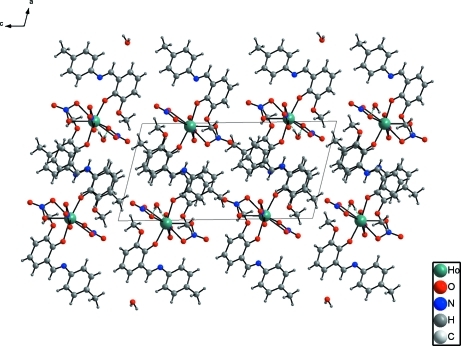
The stacking plot of the title compound, showing H-bond interactions (dashed lines) and π···π stacking interactions.

## supplementary crystallographic information

### Comment

It has been well confirmed that Schiff bases are important in multiple fields such as chemistry and biochemistry owing to their biological activities (Zhao *et al.*, 2005). Schiff base complexes prepared by ligands from substituted *o*-vanillin have attracted considerable attention in the past decades due to the intriguing biological activities of *o*-vanillin and the convenience in Schiff bases synthesis (Burrows & Bailar, 1966). As part of our interest in theis field, we have been engaged in a major effort directed toward the development of syntheses of new analogous Schiff bases derived from *o*-vanillin and their rare metal complexes. In a few articles we have reported part of our research results (Zhao *et al.*, 2007; Xian *et al.*, 2008; Li *et al.*, 2008; Liu *et al.*, 2009). Herein, we describe the structure of a new Ho^III^ complex.

The structure of the title complex is shown in Fig. 1, and the coordination environment of Ho^III^ is shown in Fig. 2. In this complex the Ho^III^ is ten-coordinated by O atoms, six of which come from three nitrate ions and two come from the Schiff base ligands (H*L*). The H*L* ligands coordinate to the Ho^III^ ion using oxygen atoms from deprotonated phenolic hydroxyl groups. Two longer bonds are provided by the two methoxy groups. The ten Ho—O bond distances are listed in Table 1 (including weak Ho—O interactions). The distances between Ho^III^ and methoxy O atoms (2.803Å and 2.740Å for Ho—O1 and Ho—O4) are shorter than similar reported complexes (Liu *et al.*, 2009), and even shorter than the distances between Ho and nitrate N, indicating their interactions are strong. The distances Ho—O(nitrate) bonds are in the range 2.388–2.522 Å. In contrast, in the Yb^III^ complex (Liu *et al.*, 2009), the Yb—O (methoxy) bonds are longer and weaker (2.833Å and 2.927 Å), which can be attributed to the ionic radii increase from Ho^III^ to Yb^III^ due to the lanthanide contraction.

The hydrogen bonds and π···π weak non-covalent interactions lend stability to the structure. The hydrogen bonds are listed in Table 2 and the stacking plot of this compound is shown in Fig. 3. Complex molecules are linked in a line through water molecules by hydrogen bonds and different lines are interlocked with benzene rings of Schiff base using π···π stacking. In H*L* ligands, the proton of the phenolic hydroxyl group is considered to have transferred to *N*-imine atom, which involving in an intramolecular hydrogen bond (Table 2).

### Experimental

Reagents and solvents used were of commercially available quality and used without further purification. The Schiff base ligand 2-[(4- methylphenyl)iminomethyl]-6-methoxy-phenol was prepared by condensation of *o*-vanillin and *p*-methylaniline with a high yield and was purified by recrystallization in ethanol. The compound was obtained by adding Ho_2_O_3_ (1 mmol, dissolved in methanol) to *N*-salicylidene-*p*-toluidine (3 mmol) in methanol solution. The mixture solution was stirred at room temperature for 8 h to obtain a purplish red solution. At last, the deposit was filtered out and the solution was kept for evaporating. The orange crystal was formed after several days.

### Refinement

The structure was solved by direct methods and successive Fourier difference synthesis. The H atoms bonded to C and N atoms were positioned geometrically and refined using a riding model [aliphatic C—H =0.96 Å (*U*_iso_(H) = 1.5*U*_eq_(C)), aromatic C—H = 0.93 Å (*U*_iso_(H) = 1.2*U*_eq_(C)) and N—H = 0.86 Å with *U*_iso_(H) = 1.2*U*_eq_(N). The H atoms bonded to water O atoms were located in difference Fourier maps and refined with O—H distance restraints of 0.83 (2) and *U*_iso_(H) = 1.5*U*_eq_(O).

### Crystal data

**Table:**

[Ho(NO_3_)_3_(C_15_H_15_NO_2_)_2_]·H_2_O	*Z* = 2
*M**_r_* = 851.54	*F*(000) = 852
Triclinic, *P*1	*D*_x_ = 1.712 Mg m^−^^3^
Hall symbol: -P 1	Mo *K*α radiation, λ = 0.71073 Å
*a* = 9.7646 (4) Å	Cell parameters from 9964 reflections
*b* = 9.9813 (4) Å	θ = 2.2–25.0°
*c* = 18.4281 (11) Å	µ = 2.47 mm^−^^1^
α = 97.862 (3)°	*T* = 296 K
β = 101.688 (3)°	Block, orange
γ = 106.270 (2)°	0.3 × 0.2 × 0.1 mm
*V* = 1652.21 (14) Å^3^	

### Data collection

**Table:**

Bruker APEXII area-detector diffractometer	5775 independent reflections
Radiation source: fine-focus sealed tube	5426 reflections with *I* > 2σ(*I*)
graphite	*R*_int_ = 0.025
Detector resolution: 8.3 pixels mm^-1^	θ_max_ = 25.0°, θ_min_ = 2.2°
φ and ω scans	*h* = −11→11
Absorption correction: multi-scan (*SADABS*; Sheldrick, 1996)	*k* = −11→11
*T*_min_ = 0.558, *T*_max_ = 0.781	*l* = −21→21
20799 measured reflections	

### Refinement

**Table:**

Refinement on *F*^2^	Primary atom site location: structure-invariant direct methods
Least-squares matrix: full	Secondary atom site location: difference Fourier map
*R*[*F*^2^ > 2σ(*F*^2^)] = 0.021	Hydrogen site location: inferred from neighbouring sites
*wR*(*F*^2^) = 0.059	H-atom parameters constrained
*S* = 1.05	*w* = 1/[σ^2^(*F*_o_^2^) + (0.0353*P*)^2^ + 0.7101*P*] where *P* = (*F*_o_^2^ + 2*F*_c_^2^)/3
5775 reflections	(Δ/σ)_max_ = 0.002
455 parameters	Δρ_max_ = 0.53 e Å^−^^3^
0 restraints	Δρ_min_ = −0.66 e Å^−^^3^

### Special details

**Table:**

Geometry. All e.s.d.'s (except the e.s.d. in the dihedral angle between two l.s. planes) are estimated using the full covariance matrix. The cell e.s.d.'s are taken into account individually in the estimation of e.s.d.'s in distances, angles and torsion angles; correlations between e.s.d.'s in cell parameters are only used when they are defined by crystal symmetry. An approximate (isotropic) treatment of cell e.s.d.'s is used for estimating e.s.d.'s involving l.s. planes.
Refinement. Refinement of *F*^2^ against ALL reflections. The weighted *R*-factor *wR* and goodness of fit *S* are based on *F*^2^, conventional *R*-factors *R* are based on *F*, with *F* set to zero for negative *F*^2^. The threshold expression of *F*^2^ > σ(*F*^2^) is used only for calculating *R*-factors(gt) *etc*. and is not relevant to the choice of reflections for refinement. *R*-factors based on *F*^2^ are statistically about twice as large as those based on *F*, and *R*- factors based on ALL data will be even larger.

### Fractional atomic coordinates and isotropic or equivalent isotropic displacement parameters (Å^2^)

**Table:**

	*x*	*y*	*z*	*U*_iso_*/*U*_eq_	
Ho1	0.032728 (12)	0.190747 (11)	0.250433 (7)	0.03820 (6)	
O1	0.1169 (2)	0.0481 (2)	0.13382 (13)	0.0576 (6)	
O1W	0.8410 (5)	0.5859 (5)	0.1785 (3)	0.1571 (18)	
H1WA	0.8790	0.5153	0.1764	0.236*	
H1WB	0.9173	0.6528	0.2097	0.236*	
N1	0.4860 (2)	0.5264 (2)	0.27705 (13)	0.0429 (5)	
H111	0.3986	0.4803	0.2792	0.051*	
C1	−0.7960 (5)	−0.5809 (4)	0.0191 (2)	0.0840 (12)	
H1B	−0.8988	−0.5983	0.0166	0.126*	
H1C	−0.7661	−0.6576	0.0359	0.126*	
H1D	−0.7799	−0.5749	−0.0302	0.126*	
O2	0.2460 (2)	0.3010 (2)	0.22126 (12)	0.0488 (5)	
N2	−0.4577 (2)	−0.0518 (2)	0.22195 (13)	0.0429 (5)	
H222	−0.3694	−0.0179	0.2172	0.051*	
C2	−0.7585 (4)	−0.3897 (3)	0.1328 (2)	0.0600 (8)	
H2	−0.8496	−0.4412	0.1387	0.072*	
O3	−0.2055 (2)	0.1476 (2)	0.25753 (11)	0.0463 (5)	
N3	0.1560 (3)	0.2742 (3)	0.41019 (16)	0.0543 (7)	
C3	−0.7064 (4)	−0.4420 (3)	0.0742 (2)	0.0583 (8)	
O4	−0.0393 (2)	0.4044 (2)	0.32834 (13)	0.0542 (5)	
N4	−0.1212 (3)	0.2407 (3)	0.11050 (15)	0.0559 (7)	
C4	−0.5716 (4)	−0.3636 (4)	0.0673 (2)	0.0682 (10)	
H4	−0.5345	−0.3975	0.0285	0.082*	
O5	0.2029 (4)	0.3157 (3)	0.47773 (14)	0.0887 (9)	
N5	0.0696 (4)	−0.0759 (3)	0.27028 (17)	0.0607 (7)	
C5	−0.4893 (4)	−0.2356 (4)	0.1164 (2)	0.0618 (9)	
H5	−0.3980	−0.1843	0.1107	0.074*	
O6	0.0375 (2)	0.1711 (2)	0.38113 (12)	0.0540 (5)	
C6	−0.5439 (3)	−0.1848 (3)	0.17382 (16)	0.0426 (6)	
O7	0.2202 (2)	0.3316 (2)	0.36292 (13)	0.0549 (5)	
C7	−0.6776 (3)	−0.2625 (3)	0.18245 (19)	0.0536 (7)	
H7	−0.7136	−0.2293	0.2219	0.064*	
O8	−0.1796 (3)	0.2690 (3)	0.05278 (15)	0.0806 (8)	
C8	−0.4950 (3)	0.0250 (3)	0.27212 (16)	0.0439 (6)	
H8	−0.5882	−0.0098	0.2803	0.053*	
O9	−0.1354 (3)	0.1161 (3)	0.11876 (14)	0.0641 (6)	
C9	−0.4003 (3)	0.1600 (3)	0.31529 (16)	0.0413 (6)	
O10	−0.0408 (3)	0.3393 (3)	0.16740 (14)	0.0634 (6)	
C10	−0.4509 (3)	0.2397 (3)	0.36775 (18)	0.0501 (7)	
H10	−0.5448	0.2019	0.3746	0.060*	
O11	0.0788 (4)	−0.1926 (3)	0.2780 (2)	0.0990 (10)	
C11	−0.3641 (4)	0.3698 (3)	0.40763 (19)	0.0561 (8)	
H11	−0.3973	0.4198	0.4429	0.067*	
O12	−0.0555 (2)	−0.0614 (2)	0.24037 (14)	0.0576 (6)	
C12	−0.2232 (4)	0.4306 (3)	0.39622 (17)	0.0518 (7)	
H12	−0.1648	0.5214	0.4231	0.062*	
O13	0.1781 (3)	0.0331 (3)	0.28993 (15)	0.0627 (6)	
C13	−0.1728 (3)	0.3566 (3)	0.34584 (16)	0.0433 (6)	
C14	−0.2581 (3)	0.2170 (3)	0.30408 (15)	0.0393 (6)	
C15	0.0485 (4)	0.5489 (4)	0.3605 (3)	0.0801 (12)	
H15A	−0.0109	0.6100	0.3520	0.120*	
H15B	0.1289	0.5748	0.3372	0.120*	
H15C	0.0866	0.5590	0.4139	0.120*	
C16	0.7697 (5)	1.0471 (4)	0.4971 (2)	0.0764 (11)	
H16A	0.8723	1.0568	0.5143	0.115*	
H16B	0.7254	1.0436	0.5391	0.115*	
H16C	0.7601	1.1273	0.4755	0.115*	
C17	0.6933 (4)	0.9118 (3)	0.43819 (19)	0.0548 (8)	
C18	0.5595 (4)	0.8179 (4)	0.4393 (2)	0.0661 (9)	
H18	0.5136	0.8405	0.4766	0.079*	
C19	0.4920 (4)	0.6914 (4)	0.3863 (2)	0.0603 (8)	
H19	0.4026	0.6296	0.3886	0.072*	
C20	0.5581 (3)	0.6576 (3)	0.33011 (17)	0.0438 (6)	
C21	0.6907 (3)	0.7501 (3)	0.32734 (18)	0.0517 (7)	
H21	0.7353	0.7286	0.2892	0.062*	
C22	0.7568 (4)	0.8744 (3)	0.38118 (19)	0.0544 (7)	
H22	0.8470	0.9351	0.3792	0.065*	
C23	0.5374 (3)	0.4682 (3)	0.22570 (17)	0.0463 (7)	
H23	0.6305	0.5178	0.2213	0.056*	
C24	0.4619 (3)	0.3351 (3)	0.17619 (16)	0.0431 (6)	
C25	0.5347 (4)	0.2803 (4)	0.1256 (2)	0.0606 (8)	
H25	0.6284	0.3345	0.1244	0.073*	
C26	0.4695 (4)	0.1506 (4)	0.0794 (2)	0.0655 (9)	
H26	0.5189	0.1161	0.0469	0.079*	
C27	0.3283 (4)	0.0675 (3)	0.08004 (18)	0.0536 (7)	
H27	0.2841	−0.0218	0.0481	0.064*	
C28	0.2556 (3)	0.1182 (3)	0.12795 (16)	0.0440 (6)	
C29	0.3184 (3)	0.2540 (3)	0.17693 (15)	0.0396 (6)	
C30	0.0412 (4)	−0.0894 (3)	0.0856 (2)	0.0599 (8)	
H30A	0.0322	−0.0814	0.0337	0.090*	
H30B	−0.0552	−0.1256	0.0939	0.090*	
H30C	0.0959	−0.1535	0.0971	0.090*	

### Atomic displacement parameters (Å^2^)

**Table:**

	*U*^11^	*U*^22^	*U*^33^	*U*^12^	*U*^13^	*U*^23^
Ho1	0.02926 (8)	0.03535 (8)	0.04532 (9)	0.00330 (5)	0.01079 (6)	0.00560 (5)
O1	0.0416 (11)	0.0540 (12)	0.0632 (14)	−0.0006 (10)	0.0172 (10)	−0.0069 (10)
O1W	0.123 (3)	0.135 (4)	0.193 (5)	0.017 (3)	0.047 (3)	0.008 (3)
N1	0.0330 (11)	0.0453 (12)	0.0471 (13)	0.0060 (10)	0.0115 (10)	0.0104 (10)
C1	0.092 (3)	0.051 (2)	0.077 (3)	0.000 (2)	−0.008 (2)	−0.0038 (18)
O2	0.0399 (10)	0.0468 (11)	0.0582 (13)	0.0073 (9)	0.0236 (9)	0.0034 (9)
N2	0.0319 (11)	0.0442 (12)	0.0471 (13)	0.0067 (10)	0.0089 (10)	0.0044 (10)
C2	0.0469 (18)	0.0458 (16)	0.076 (2)	0.0018 (14)	0.0081 (16)	0.0121 (16)
O3	0.0337 (10)	0.0489 (11)	0.0516 (12)	0.0096 (8)	0.0138 (9)	−0.0018 (9)
N3	0.0600 (17)	0.0523 (15)	0.0489 (16)	0.0259 (14)	0.0057 (13)	0.0001 (12)
C3	0.060 (2)	0.0410 (15)	0.058 (2)	0.0056 (15)	−0.0045 (16)	0.0090 (14)
O4	0.0412 (11)	0.0450 (11)	0.0667 (14)	0.0009 (9)	0.0135 (10)	0.0073 (10)
N4	0.0475 (15)	0.081 (2)	0.0475 (16)	0.0273 (15)	0.0177 (13)	0.0160 (14)
C4	0.078 (2)	0.0536 (19)	0.062 (2)	0.0093 (18)	0.0233 (19)	−0.0056 (16)
O5	0.122 (2)	0.0850 (19)	0.0443 (15)	0.0339 (18)	−0.0016 (15)	−0.0045 (13)
N5	0.084 (2)	0.0528 (16)	0.0702 (19)	0.0367 (16)	0.0435 (16)	0.0253 (14)
C5	0.0524 (19)	0.0562 (19)	0.067 (2)	0.0031 (15)	0.0231 (17)	−0.0011 (16)
O6	0.0557 (13)	0.0512 (12)	0.0545 (13)	0.0116 (11)	0.0193 (10)	0.0120 (10)
C6	0.0375 (14)	0.0402 (14)	0.0436 (15)	0.0073 (12)	0.0037 (12)	0.0070 (12)
O7	0.0391 (11)	0.0573 (12)	0.0570 (13)	0.0042 (10)	0.0116 (10)	−0.0009 (10)
C7	0.0484 (17)	0.0487 (16)	0.060 (2)	0.0093 (14)	0.0151 (15)	0.0101 (14)
O8	0.0852 (18)	0.118 (2)	0.0553 (15)	0.0523 (17)	0.0167 (13)	0.0323 (15)
C8	0.0343 (14)	0.0505 (16)	0.0467 (16)	0.0136 (12)	0.0109 (12)	0.0085 (13)
O9	0.0726 (16)	0.0697 (16)	0.0537 (14)	0.0343 (13)	0.0126 (12)	0.0048 (11)
C9	0.0363 (14)	0.0445 (14)	0.0430 (15)	0.0134 (12)	0.0093 (12)	0.0084 (12)
O10	0.0539 (13)	0.0643 (14)	0.0691 (16)	0.0104 (11)	0.0129 (12)	0.0261 (12)
C10	0.0438 (16)	0.0590 (18)	0.0512 (17)	0.0193 (14)	0.0180 (14)	0.0087 (14)
O11	0.143 (3)	0.0614 (16)	0.132 (3)	0.0567 (18)	0.070 (2)	0.0455 (17)
C11	0.064 (2)	0.0538 (18)	0.0557 (19)	0.0268 (16)	0.0213 (16)	0.0013 (15)
O12	0.0578 (13)	0.0410 (11)	0.0769 (16)	0.0092 (10)	0.0330 (12)	0.0109 (10)
C12	0.0572 (18)	0.0426 (15)	0.0493 (17)	0.0160 (14)	0.0056 (14)	0.0006 (13)
O13	0.0536 (13)	0.0584 (14)	0.0823 (17)	0.0222 (12)	0.0222 (12)	0.0184 (12)
C13	0.0376 (14)	0.0412 (14)	0.0490 (16)	0.0105 (12)	0.0079 (12)	0.0107 (12)
C14	0.0361 (13)	0.0417 (14)	0.0405 (15)	0.0140 (12)	0.0083 (11)	0.0082 (11)
C15	0.064 (2)	0.0429 (18)	0.126 (4)	0.0051 (17)	0.024 (2)	0.018 (2)
C16	0.080 (3)	0.066 (2)	0.068 (2)	0.014 (2)	0.010 (2)	−0.0016 (18)
C17	0.0561 (19)	0.0502 (17)	0.0542 (19)	0.0169 (15)	0.0054 (15)	0.0103 (14)
C18	0.059 (2)	0.075 (2)	0.063 (2)	0.0185 (18)	0.0241 (17)	0.0003 (18)
C19	0.0449 (17)	0.065 (2)	0.066 (2)	0.0076 (15)	0.0227 (16)	0.0065 (17)
C20	0.0383 (14)	0.0441 (14)	0.0480 (16)	0.0103 (12)	0.0103 (12)	0.0134 (12)
C21	0.0481 (17)	0.0509 (16)	0.0527 (18)	0.0066 (14)	0.0177 (14)	0.0113 (14)
C22	0.0486 (17)	0.0489 (16)	0.0577 (19)	0.0024 (14)	0.0130 (15)	0.0134 (14)
C23	0.0383 (15)	0.0494 (16)	0.0546 (18)	0.0114 (13)	0.0177 (13)	0.0186 (13)
C24	0.0395 (14)	0.0468 (15)	0.0460 (16)	0.0138 (12)	0.0154 (12)	0.0121 (12)
C25	0.0513 (18)	0.066 (2)	0.070 (2)	0.0146 (16)	0.0346 (17)	0.0114 (17)
C26	0.067 (2)	0.064 (2)	0.072 (2)	0.0207 (18)	0.0410 (19)	0.0033 (18)
C27	0.0568 (18)	0.0526 (17)	0.0520 (18)	0.0170 (15)	0.0202 (15)	0.0034 (14)
C28	0.0407 (15)	0.0485 (15)	0.0429 (16)	0.0127 (13)	0.0110 (12)	0.0123 (12)
C29	0.0360 (14)	0.0446 (14)	0.0411 (15)	0.0131 (12)	0.0127 (12)	0.0135 (12)
C30	0.0573 (19)	0.0480 (17)	0.060 (2)	0.0055 (15)	0.0093 (16)	−0.0027 (15)

### Geometric parameters (Å, °)

**Table:**

Ho1—O2	2.2734 (18)	C5—H5	0.9300
Ho1—O3	2.2787 (18)	C6—C7	1.371 (4)
Ho1—O12	2.388 (2)	C7—H7	0.9300
Ho1—O10	2.414 (2)	C8—C9	1.419 (4)
Ho1—O7	2.418 (2)	C8—H8	0.9300
Ho1—O6	2.435 (2)	C9—C14	1.414 (4)
Ho1—O13	2.483 (2)	C9—C10	1.420 (4)
Ho1—O9	2.519 (2)	C10—C11	1.347 (4)
Ho1—O4	2.742 (2)	C10—H10	0.9300
Ho1—O1	2.803 (2)	C11—C12	1.410 (5)
Ho1—N5	2.846 (3)	C11—H11	0.9300
Ho1—N3	2.853 (3)	C12—C13	1.361 (4)
O1—C28	1.373 (3)	C12—H12	0.9300
O1—C30	1.432 (4)	C13—C14	1.423 (4)
O1W—H1WA	0.8833	C15—H15A	0.9600
O1W—H1WB	0.8797	C15—H15B	0.9600
N1—C23	1.300 (4)	C15—H15C	0.9600
N1—C20	1.422 (4)	C16—C17	1.506 (5)
N1—H111	0.8600	C16—H16A	0.9600
C1—C3	1.514 (4)	C16—H16B	0.9600
C1—H1B	0.9600	C16—H16C	0.9600
C1—H1C	0.9600	C17—C22	1.383 (5)
C1—H1D	0.9600	C17—C18	1.384 (5)
O2—C29	1.304 (3)	C18—C19	1.385 (5)
N2—C8	1.294 (4)	C18—H18	0.9300
N2—C6	1.417 (3)	C19—C20	1.379 (4)
N2—H222	0.8600	C19—H19	0.9300
C2—C7	1.379 (4)	C20—C21	1.379 (4)
C2—C3	1.383 (5)	C21—C22	1.376 (4)
C2—H2	0.9300	C21—H21	0.9300
O3—C14	1.302 (3)	C22—H22	0.9300
N3—O5	1.205 (4)	C23—C24	1.410 (4)
N3—O6	1.271 (3)	C23—H23	0.9300
N3—O7	1.279 (4)	C24—C29	1.412 (4)
C3—C4	1.371 (5)	C24—C25	1.421 (4)
O4—C13	1.377 (3)	C25—C26	1.351 (5)
O4—C15	1.425 (4)	C25—H25	0.9300
N4—O8	1.210 (4)	C26—C27	1.399 (5)
N4—O9	1.246 (4)	C26—H26	0.9300
N4—O10	1.279 (4)	C27—C28	1.369 (4)
C4—C5	1.382 (5)	C27—H27	0.9300
C4—H4	0.9300	C28—C29	1.415 (4)
N5—O11	1.219 (4)	C30—H30A	0.9600
N5—O13	1.236 (4)	C30—H30B	0.9600
N5—O12	1.290 (4)	C30—H30C	0.9600
C5—C6	1.376 (4)		
			
O2—Ho1—O3	157.35 (8)	O10—N4—Ho1	55.70 (15)
O2—Ho1—O12	122.32 (7)	C3—C4—C5	121.9 (3)
O3—Ho1—O12	76.29 (7)	C3—C4—H4	119.0
O2—Ho1—O10	77.14 (8)	C5—C4—H4	119.0
O3—Ho1—O10	80.66 (8)	O11—N5—O13	122.2 (3)
O12—Ho1—O10	130.72 (8)	O11—N5—O12	120.8 (3)
O2—Ho1—O7	68.94 (7)	O13—N5—O12	117.0 (2)
O3—Ho1—O7	116.17 (7)	O11—N5—Ho1	177.2 (3)
O12—Ho1—O7	117.76 (8)	O13—N5—Ho1	60.54 (15)
O10—Ho1—O7	111.47 (8)	O12—N5—Ho1	56.43 (13)
O2—Ho1—O6	119.21 (8)	C6—C5—C4	119.3 (3)
O3—Ho1—O6	74.87 (7)	C6—C5—H5	120.4
O12—Ho1—O6	77.71 (8)	C4—C5—H5	120.4
O10—Ho1—O6	136.05 (8)	N3—O6—Ho1	95.53 (17)
O7—Ho1—O6	52.74 (7)	C7—C6—C5	119.8 (3)
O2—Ho1—O13	80.46 (8)	C7—C6—N2	122.6 (3)
O3—Ho1—O13	122.10 (8)	C5—C6—N2	117.6 (3)
O12—Ho1—O13	52.45 (8)	N3—O7—Ho1	96.12 (16)
O10—Ho1—O13	152.22 (9)	C6—C7—C2	120.1 (3)
O7—Ho1—O13	74.71 (8)	C6—C7—H7	119.9
O6—Ho1—O13	70.04 (8)	C2—C7—H7	119.9
O2—Ho1—O9	98.42 (8)	N2—C8—C9	123.1 (3)
O3—Ho1—O9	70.56 (8)	N2—C8—H8	118.5
O12—Ho1—O9	79.83 (9)	C9—C8—H8	118.5
O10—Ho1—O9	51.40 (8)	N4—O9—Ho1	94.22 (18)
O7—Ho1—O9	161.82 (9)	C14—C9—C8	120.1 (2)
O6—Ho1—O9	142.26 (8)	C14—C9—C10	120.2 (3)
O13—Ho1—O9	117.22 (8)	C8—C9—C10	119.7 (3)
O2—Ho1—O4	105.43 (6)	N4—O10—Ho1	98.35 (18)
O3—Ho1—O4	61.81 (6)	C11—C10—C9	120.5 (3)
O12—Ho1—O4	130.33 (7)	C11—C10—H10	119.7
O10—Ho1—O4	69.31 (8)	C9—C10—H10	119.7
O7—Ho1—O4	65.47 (7)	C10—C11—C12	120.5 (3)
O6—Ho1—O4	67.02 (7)	C10—C11—H11	119.8
O13—Ho1—O4	133.42 (8)	C12—C11—H11	119.8
O9—Ho1—O4	107.68 (7)	N5—O12—Ho1	96.81 (17)
O2—Ho1—O1	60.97 (6)	C13—C12—C11	120.0 (3)
O3—Ho1—O1	123.71 (6)	C13—C12—H12	120.0
O12—Ho1—O1	69.08 (7)	C11—C12—H12	120.0
O10—Ho1—O1	89.68 (8)	N5—O13—Ho1	93.77 (18)
O7—Ho1—O1	118.91 (7)	C12—C13—O4	125.9 (3)
O6—Ho1—O1	134.27 (7)	C12—C13—C14	121.8 (3)
O13—Ho1—O1	64.94 (8)	O4—C13—C14	112.3 (2)
O9—Ho1—O1	60.84 (7)	O3—C14—C9	122.7 (2)
O4—Ho1—O1	157.72 (7)	O3—C14—C13	120.3 (2)
O2—Ho1—N5	101.59 (8)	C9—C14—C13	117.0 (2)
O3—Ho1—N5	99.79 (8)	O4—C15—H15A	109.5
O12—Ho1—N5	26.76 (8)	O4—C15—H15B	109.5
O10—Ho1—N5	149.46 (9)	H15A—C15—H15B	109.5
O7—Ho1—N5	95.87 (9)	O4—C15—H15C	109.5
O6—Ho1—N5	71.66 (8)	H15A—C15—H15C	109.5
O13—Ho1—N5	25.69 (8)	H15B—C15—H15C	109.5
O9—Ho1—N5	99.58 (9)	C17—C16—H16A	109.5
O4—Ho1—N5	137.82 (7)	C17—C16—H16B	109.5
O1—Ho1—N5	64.44 (8)	H16A—C16—H16B	109.5
O2—Ho1—N3	94.50 (8)	C17—C16—H16C	109.5
O3—Ho1—N3	95.05 (8)	H16A—C16—H16C	109.5
O12—Ho1—N3	98.74 (8)	H16B—C16—H16C	109.5
O10—Ho1—N3	126.41 (8)	C22—C17—C18	117.0 (3)
O7—Ho1—N3	26.47 (8)	C22—C17—C16	120.8 (3)
O6—Ho1—N3	26.32 (8)	C18—C17—C16	122.1 (3)
O13—Ho1—N3	71.33 (8)	C19—C18—C17	121.9 (3)
O9—Ho1—N3	165.53 (8)	C19—C18—H18	119.1
O4—Ho1—N3	62.21 (7)	C17—C18—H18	119.1
O1—Ho1—N3	132.29 (7)	C20—C19—C18	119.6 (3)
N5—Ho1—N3	84.10 (8)	C20—C19—H19	120.2
C28—O1—C30	117.5 (2)	C18—C19—H19	120.2
C28—O1—Ho1	113.27 (16)	C19—C20—C21	119.6 (3)
C30—O1—Ho1	129.00 (18)	C19—C20—N1	118.0 (3)
H1WA—O1W—H1WB	98.7	C21—C20—N1	122.4 (3)
C23—N1—C20	127.3 (2)	C22—C21—C20	119.8 (3)
C23—N1—H111	116.4	C22—C21—H21	120.1
C20—N1—H111	116.4	C20—C21—H21	120.1
C3—C1—H1B	109.5	C21—C22—C17	122.1 (3)
C3—C1—H1C	109.5	C21—C22—H22	119.0
H1B—C1—H1C	109.5	C17—C22—H22	119.0
C3—C1—H1D	109.5	N1—C23—C24	124.5 (3)
H1B—C1—H1D	109.5	N1—C23—H23	117.8
H1C—C1—H1D	109.5	C24—C23—H23	117.8
C29—O2—Ho1	131.40 (17)	C23—C24—C29	121.9 (3)
C8—N2—C6	128.2 (2)	C23—C24—C25	118.6 (3)
C8—N2—H222	115.9	C29—C24—C25	119.5 (3)
C6—N2—H222	115.9	C26—C25—C24	120.8 (3)
C7—C2—C3	121.0 (3)	C26—C25—H25	119.6
C7—C2—H2	119.5	C24—C25—H25	119.6
C3—C2—H2	119.5	C25—C26—C27	120.6 (3)
C14—O3—Ho1	128.69 (17)	C25—C26—H26	119.7
O5—N3—O6	121.9 (3)	C27—C26—H26	119.7
O5—N3—O7	122.7 (3)	C28—C27—C26	119.7 (3)
O6—N3—O7	115.4 (2)	C28—C27—H27	120.2
O5—N3—Ho1	175.7 (2)	C26—C27—H27	120.2
O6—N3—Ho1	58.15 (14)	C27—C28—O1	125.5 (3)
O7—N3—Ho1	57.42 (14)	C27—C28—C29	121.9 (3)
C4—C3—C2	117.8 (3)	O1—C28—C29	112.6 (2)
C4—C3—C1	121.1 (4)	O2—C29—C24	121.8 (3)
C2—C3—C1	121.1 (3)	O2—C29—C28	120.8 (2)
C13—O4—C15	117.2 (3)	C24—C29—C28	117.4 (2)
C13—O4—Ho1	112.94 (16)	O1—C30—H30A	109.5
C15—O4—Ho1	129.3 (2)	O1—C30—H30B	109.5
O8—N4—O9	123.0 (3)	H30A—C30—H30B	109.5
O8—N4—O10	121.0 (3)	O1—C30—H30C	109.5
O9—N4—O10	116.0 (3)	H30A—C30—H30C	109.5
O8—N4—Ho1	176.4 (3)	H30B—C30—H30C	109.5
O9—N4—Ho1	60.32 (16)		
			
O2—Ho1—O1—C28	7.49 (18)	O3—Ho1—O6—N3	−138.80 (17)
O3—Ho1—O1—C28	161.46 (18)	O12—Ho1—O6—N3	142.28 (17)
O12—Ho1—O1—C28	−142.6 (2)	O10—Ho1—O6—N3	−80.32 (18)
O10—Ho1—O1—C28	82.9 (2)	O7—Ho1—O6—N3	2.42 (15)
O7—Ho1—O1—C28	−31.6 (2)	O13—Ho1—O6—N3	88.05 (16)
O6—Ho1—O1—C28	−96.3 (2)	O9—Ho1—O6—N3	−162.94 (15)
O13—Ho1—O1—C28	−85.4 (2)	O4—Ho1—O6—N3	−73.45 (16)
O9—Ho1—O1—C28	127.6 (2)	O1—Ho1—O6—N3	98.56 (16)
O4—Ho1—O1—C28	64.0 (3)	N5—Ho1—O6—N3	115.23 (17)
N5—Ho1—O1—C28	−113.9 (2)	C4—C5—C6—C7	1.1 (5)
N3—Ho1—O1—C28	−59.9 (2)	C4—C5—C6—N2	−178.7 (3)
O2—Ho1—O1—C30	−178.2 (3)	C8—N2—C6—C7	−10.2 (5)
O3—Ho1—O1—C30	−24.2 (3)	C8—N2—C6—C5	169.6 (3)
O12—Ho1—O1—C30	31.7 (3)	O5—N3—O7—Ho1	−174.8 (3)
O10—Ho1—O1—C30	−102.8 (3)	O6—N3—O7—Ho1	4.1 (2)
O7—Ho1—O1—C30	142.7 (3)	O2—Ho1—O7—N3	−164.19 (18)
O6—Ho1—O1—C30	78.0 (3)	O3—Ho1—O7—N3	39.94 (18)
O13—Ho1—O1—C30	88.9 (3)	O12—Ho1—O7—N3	−47.79 (18)
O9—Ho1—O1—C30	−58.1 (3)	O10—Ho1—O7—N3	129.87 (16)
O4—Ho1—O1—C30	−121.7 (3)	O6—Ho1—O7—N3	−2.41 (14)
N5—Ho1—O1—C30	60.5 (3)	O13—Ho1—O7—N3	−78.72 (16)
N3—Ho1—O1—C30	114.4 (3)	O9—Ho1—O7—N3	147.9 (2)
O3—Ho1—O2—C29	−117.7 (3)	O4—Ho1—O7—N3	76.52 (16)
O12—Ho1—O2—C29	24.3 (3)	O1—Ho1—O7—N3	−127.98 (15)
O10—Ho1—O2—C29	−106.0 (2)	N5—Ho1—O7—N3	−64.00 (17)
O7—Ho1—O2—C29	134.6 (3)	C5—C6—C7—C2	−1.4 (5)
O6—Ho1—O2—C29	118.0 (2)	N2—C6—C7—C2	178.4 (3)
O13—Ho1—O2—C29	57.4 (2)	C3—C2—C7—C6	0.7 (5)
O9—Ho1—O2—C29	−58.9 (2)	C6—N2—C8—C9	−177.6 (3)
O4—Ho1—O2—C29	−170.0 (2)	O8—N4—O9—Ho1	178.2 (3)
O1—Ho1—O2—C29	−9.1 (2)	O10—N4—O9—Ho1	−2.3 (3)
N5—Ho1—O2—C29	42.7 (3)	O2—Ho1—O9—N4	−64.67 (18)
N3—Ho1—O2—C29	127.6 (2)	O3—Ho1—O9—N4	94.90 (18)
O2—Ho1—O3—C14	−77.2 (3)	O12—Ho1—O9—N4	173.83 (19)
O12—Ho1—O3—C14	135.2 (2)	O10—Ho1—O9—N4	1.37 (16)
O10—Ho1—O3—C14	−88.7 (2)	O7—Ho1—O9—N4	−20.2 (3)
O7—Ho1—O3—C14	20.7 (3)	O6—Ho1—O9—N4	119.64 (19)
O6—Ho1—O3—C14	54.4 (2)	O13—Ho1—O9—N4	−148.22 (16)
O13—Ho1—O3—C14	108.5 (2)	O4—Ho1—O9—N4	44.57 (19)
O9—Ho1—O3—C14	−141.0 (2)	O1—Ho1—O9—N4	−114.55 (19)
O4—Ho1—O3—C14	−17.3 (2)	N5—Ho1—O9—N4	−168.02 (17)
O1—Ho1—O3—C14	−172.0 (2)	N3—Ho1—O9—N4	88.3 (4)
N5—Ho1—O3—C14	122.3 (2)	N2—C8—C9—C14	0.5 (4)
N3—Ho1—O3—C14	37.4 (2)	N2—C8—C9—C10	178.2 (3)
O2—Ho1—N3—O6	−160.90 (16)	O8—N4—O10—Ho1	−178.1 (2)
O3—Ho1—N3—O6	39.66 (16)	O9—N4—O10—Ho1	2.4 (3)
O12—Ho1—N3—O6	−37.21 (17)	O2—Ho1—O10—N4	110.64 (18)
O10—Ho1—N3—O6	121.78 (16)	O3—Ho1—O10—N4	−73.88 (17)
O7—Ho1—N3—O6	−175.7 (3)	O12—Ho1—O10—N4	−11.2 (2)
O13—Ho1—N3—O6	−82.56 (16)	O7—Ho1—O10—N4	171.57 (16)
O9—Ho1—N3—O6	45.9 (4)	O6—Ho1—O10—N4	−130.39 (17)
O4—Ho1—N3—O6	94.02 (17)	O13—Ho1—O10—N4	73.6 (2)
O1—Ho1—N3—O6	−106.81 (17)	O9—Ho1—O10—N4	−1.35 (16)
N5—Ho1—N3—O6	−59.68 (16)	O4—Ho1—O10—N4	−137.16 (18)
O2—Ho1—N3—O7	14.77 (17)	O1—Ho1—O10—N4	50.41 (17)
O3—Ho1—N3—O7	−144.66 (16)	N5—Ho1—O10—N4	19.6 (3)
O12—Ho1—N3—O7	138.46 (16)	N3—Ho1—O10—N4	−163.29 (16)
O10—Ho1—N3—O7	−62.55 (19)	C14—C9—C10—C11	−0.4 (5)
O6—Ho1—N3—O7	175.7 (3)	C8—C9—C10—C11	−178.1 (3)
O13—Ho1—N3—O7	93.11 (17)	C9—C10—C11—C12	1.9 (5)
O9—Ho1—N3—O7	−138.4 (3)	O11—N5—O12—Ho1	179.3 (3)
O4—Ho1—N3—O7	−90.31 (17)	O13—N5—O12—Ho1	−1.1 (3)
O1—Ho1—N3—O7	68.86 (18)	O2—Ho1—O12—N5	43.4 (2)
N5—Ho1—N3—O7	115.99 (17)	O3—Ho1—O12—N5	−150.72 (18)
C7—C2—C3—C4	0.2 (5)	O10—Ho1—O12—N5	144.77 (17)
C7—C2—C3—C1	−179.3 (3)	O7—Ho1—O12—N5	−38.10 (19)
O2—Ho1—O4—C13	175.66 (18)	O6—Ho1—O12—N5	−73.53 (17)
O3—Ho1—O4—C13	15.89 (17)	O13—Ho1—O12—N5	0.61 (16)
O12—Ho1—O4—C13	−20.2 (2)	O9—Ho1—O12—N5	136.99 (18)
O10—Ho1—O4—C13	106.21 (19)	O4—Ho1—O12—N5	−118.38 (17)
O7—Ho1—O4—C13	−126.7 (2)	O1—Ho1—O12—N5	74.47 (17)
O6—Ho1—O4—C13	−68.70 (18)	N3—Ho1—O12—N5	−57.61 (18)
O13—Ho1—O4—C13	−92.95 (19)	C10—C11—C12—C13	−1.4 (5)
O9—Ho1—O4—C13	71.34 (19)	O11—N5—O13—Ho1	−179.4 (3)
O1—Ho1—O4—C13	126.5 (2)	O12—N5—O13—Ho1	1.0 (3)
N5—Ho1—O4—C13	−56.4 (2)	O2—Ho1—O13—N5	−145.02 (19)
N3—Ho1—O4—C13	−97.41 (19)	O3—Ho1—O13—N5	32.8 (2)
O2—Ho1—O4—C15	−13.3 (3)	O12—Ho1—O13—N5	−0.63 (17)
O3—Ho1—O4—C15	−173.1 (3)	O10—Ho1—O13—N5	−108.5 (2)
O12—Ho1—O4—C15	150.8 (3)	O7—Ho1—O13—N5	144.4 (2)
O10—Ho1—O4—C15	−82.8 (3)	O6—Ho1—O13—N5	89.00 (19)
O7—Ho1—O4—C15	44.3 (3)	O9—Ho1—O13—N5	−50.4 (2)
O6—Ho1—O4—C15	102.3 (3)	O4—Ho1—O13—N5	112.72 (18)
O13—Ho1—O4—C15	78.1 (3)	O1—Ho1—O13—N5	−82.71 (19)
O9—Ho1—O4—C15	−117.7 (3)	N3—Ho1—O13—N5	116.9 (2)
O1—Ho1—O4—C15	−62.5 (4)	C11—C12—C13—O4	178.7 (3)
N5—Ho1—O4—C15	114.6 (3)	C11—C12—C13—C14	−0.6 (5)
N3—Ho1—O4—C15	73.6 (3)	C15—O4—C13—C12	−6.1 (5)
O2—Ho1—N4—O9	116.46 (18)	Ho1—O4—C13—C12	166.1 (2)
O3—Ho1—N4—O9	−77.07 (18)	C15—O4—C13—C14	173.3 (3)
O12—Ho1—N4—O9	−6.29 (19)	Ho1—O4—C13—C14	−14.5 (3)
O10—Ho1—N4—O9	−177.5 (3)	Ho1—O3—C14—C9	−163.8 (2)
O7—Ho1—N4—O9	170.90 (16)	Ho1—O3—C14—C13	16.8 (4)
O6—Ho1—N4—O9	−97.1 (2)	C8—C9—C14—O3	−3.3 (4)
O13—Ho1—N4—O9	43.9 (2)	C10—C9—C14—O3	179.0 (3)
O4—Ho1—N4—O9	−138.03 (18)	C8—C9—C14—C13	176.1 (3)
O1—Ho1—N4—O9	55.94 (17)	C10—C9—C14—C13	−1.6 (4)
N5—Ho1—N4—O9	14.4 (2)	C12—C13—C14—O3	−178.5 (3)
N3—Ho1—N4—O9	−149.44 (19)	O4—C13—C14—O3	2.1 (4)
O2—Ho1—N4—O10	−65.99 (18)	C12—C13—C14—C9	2.1 (4)
O3—Ho1—N4—O10	100.48 (18)	O4—C13—C14—C9	−177.3 (2)
O12—Ho1—N4—O10	171.25 (17)	C22—C17—C18—C19	0.6 (5)
O7—Ho1—N4—O10	−11.6 (2)	C16—C17—C18—C19	−177.9 (4)
O6—Ho1—N4—O10	80.4 (2)	C17—C18—C19—C20	−0.8 (6)
O13—Ho1—N4—O10	−138.51 (18)	C18—C19—C20—C21	0.0 (5)
O9—Ho1—N4—O10	177.5 (3)	C18—C19—C20—N1	179.6 (3)
O4—Ho1—N4—O10	39.51 (17)	C23—N1—C20—C19	−171.7 (3)
O1—Ho1—N4—O10	−126.52 (18)	C23—N1—C20—C21	7.8 (4)
N5—Ho1—N4—O10	−168.10 (17)	C19—C20—C21—C22	0.8 (5)
N3—Ho1—N4—O10	28.1 (3)	N1—C20—C21—C22	−178.7 (3)
C2—C3—C4—C5	−0.5 (6)	C20—C21—C22—C17	−1.1 (5)
C1—C3—C4—C5	179.0 (4)	C18—C17—C22—C21	0.3 (5)
O2—Ho1—N5—O13	35.2 (2)	C16—C17—C22—C21	178.9 (3)
O3—Ho1—N5—O13	−152.29 (18)	C20—N1—C23—C24	177.9 (3)
O12—Ho1—N5—O13	178.9 (3)	N1—C23—C24—C29	2.0 (5)
O10—Ho1—N5—O13	119.5 (2)	N1—C23—C24—C25	−176.4 (3)
O7—Ho1—N5—O13	−34.41 (19)	C23—C24—C25—C26	177.1 (3)
O6—Ho1—N5—O13	−81.93 (19)	C29—C24—C25—C26	−1.3 (5)
O9—Ho1—N5—O13	135.97 (19)	C24—C25—C26—C27	0.3 (6)
O4—Ho1—N5—O13	−93.9 (2)	C25—C26—C27—C28	0.0 (6)
O1—Ho1—N5—O13	84.91 (19)	C26—C27—C28—O1	179.6 (3)
N3—Ho1—N5—O13	−58.15 (19)	C26—C27—C28—C29	0.6 (5)
O2—Ho1—N5—O12	−143.64 (17)	C30—O1—C28—C27	−0.3 (5)
O3—Ho1—N5—O12	28.83 (18)	Ho1—O1—C28—C27	174.7 (2)
O10—Ho1—N5—O12	−59.3 (2)	C30—O1—C28—C29	178.7 (3)
O7—Ho1—N5—O12	146.71 (17)	Ho1—O1—C28—C29	−6.3 (3)
O6—Ho1—N5—O12	99.18 (18)	Ho1—O2—C29—C24	−169.68 (19)
O13—Ho1—N5—O12	−178.9 (3)	Ho1—O2—C29—C28	9.7 (4)
O9—Ho1—N5—O12	−42.91 (18)	C23—C24—C29—O2	3.0 (4)
O4—Ho1—N5—O12	87.2 (2)	C25—C24—C29—O2	−178.7 (3)
O1—Ho1—N5—O12	−93.98 (18)	C23—C24—C29—C28	−176.5 (3)
N3—Ho1—N5—O12	122.96 (18)	C25—C24—C29—C28	1.9 (4)
C3—C4—C5—C6	−0.1 (6)	C27—C28—C29—O2	179.0 (3)
O5—N3—O6—Ho1	174.9 (3)	O1—C28—C29—O2	−0.1 (4)
O7—N3—O6—Ho1	−4.0 (2)	C27—C28—C29—C24	−1.6 (4)
O2—Ho1—O6—N3	21.95 (18)	O1—C28—C29—C24	179.3 (2)

### Hydrogen-bond geometry (Å, °)

**Table:**

*D*—H···*A*	*D*—H	H···*A*	*D*···*A*	*D*—H···*A*
O1W—H1WA···O10^i^	0.88	2.11	2.996 (6)	177
O1W—H1WB···O11^ii^	0.88	1.94	2.817 (6)	177
N1—H111···O2	0.86	1.98	2.655 (3)	135
N2—H222···O3	0.86	1.88	2.588 (3)	138

Symmetry codes: (i) *x*+1, *y*, *z*; (ii) *x*+1, *y*+1, *z*.

## References

[bb1] Bruker (2006). *APEX2 *and *SAINT* Bruker AXS Inc., Madison, Wisconsin, USA.

[bb2] Burrows, R. C. & Bailar, J. C. (1966). *J. Am. Chem. Soc.* **88**, 4150–4152.

[bb3] Li, H.-Q., Xian, H.-D., Liu, J.-F. & Zhao, G.-L. (2008). *Acta Cryst.* E**64**, m1593–m1594. 10.1107/S1600536808038099 PMC2959993 21581192

[bb4] Liu, J.-F., Liu, J.-L. & Zhao, G.-L. (2009). *Acta Cryst.* E**65**, m1385–m1386. 10.1107/S1600536809041361 PMC2971141 21578132

[bb5] Sheldrick, G. M. (1996). *SADABS* University of Göttingen, Germany.

[bb6] Sheldrick, G. M. (2008). *Acta Cryst.* A**64**, 112–122. 10.1107/S0108767307043930 18156677

[bb7] Xian, H.-D., Liu, J.-F., Li, H.-Q. & Zhao, G.-L. (2008). *Acta Cryst.* E**64**, m1422. 10.1107/S1600536808033102 PMC2959557 21580867

[bb8] Zhao, G.-L., Shi, X. & Ng, S. W. (2007). *Acta Cryst.* E**63**, m267–m268.

[bb9] Zhao, G.-L., Zhang, P.-H. & Feng, Y.-L. (2005). *Chin. J. Inorg. Chem.* **21**, 421–424.

